# Identification of Extremely Rare Pathogenic CNVs by Array CGH in Saudi Children with Developmental Delay, Congenital Malformations, and Intellectual Disability

**DOI:** 10.3390/children10040662

**Published:** 2023-03-31

**Authors:** Sajjad Karim, Ibtessam Ramzi Hussein, Hans-Juergen Schulten, Saad Alsaedi, Zeenat Mirza, Mohammed Al-Qahtani, Adeel Chaudhary

**Affiliations:** 1 Center of Excellence in Genomic Medicine Research, King Abdulaziz University, Jeddah 21589, Saudi Arabia; 2 Department of Medical Laboratory Science, Faculty of Applied Medical Sciences, King Abdulaziz University, Jeddah 21589, Saudi Arabia; 3 Molecular Genetics & Enzymology Department, Division of Human Genetics & Genome, National Research Centre, Dokki, Giza 12311, Egypt; 4 Department of Pediatrics, Faculty of Medicine, King Abdulaziz University, Jeddah 21589, Saudi Arabia; 5 King Fahd Medical Research Center, King Abdulaziz University, Jeddah 21589, Saudi Arabia; 6 Center of Innovation in Personalized Medicine, King Abdulaziz University, Jeddah 21589, Saudi Arabia

**Keywords:** array comparative genomic hybridization, copy number variations, developmental delay, congenital malformations, Saudi Arabia

## Abstract

Chromosomal imbalance is implicated in developmental delay (DD), congenital malformations (CM), and intellectual disability (ID), and, thus, precise identification of copy number variations (CNVs) is essential. We therefore aimed to investigate the genetic heterogeneity in Saudi children with DD/CM/ID. High-resolution array comparative genomic hybridization (array CGH) was used to detect disease-associated CNVs in 63 patients. Quantitative PCR was done to confirm the detected CNVs. Giemsa banding-based karyotyping was also performed. Array CGH identified chromosomal abnormalities in 24 patients; distinct pathogenic and/or variants of uncertain significance CNVs were found in 19 patients, and aneuploidy was found in 5 patients including 47,XXY (n = 2), 45,X (n = 2) and a patient with trisomy 18 who carried a balanced Robertsonian translocation. CNVs including 9p24p13, 16p13p11, 18p11 had gains/duplications and CNVs, including 3p23p14, 10q26, 11p15, 11q24q25, 13q21.1q32.1, 16p13.3p11.2, and 20q11.1q13.2, had losses/deletions only, while CNVs including 8q24, 11q12, 15q25q26, 16q21q23, and 22q11q13 were found with both gains or losses in different individuals. In contrast, standard karyotyping detected chromosomal abnormalities in ten patients. The diagnosis rate of array CGH (28%, 18/63 patients) was around two-fold higher than that of conventional karyotyping (15.87%, 10/63 patients). We herein report, for the first time, the extremely rare pathogenic CNVs in Saudi children with DD/CM/ID. The reported prevalence of CNVs in Saudi Arabia adds value to clinical cytogenetics.

## 1. Introduction

Children under the age of 5 years are categorized as individuals with global developmental delay (DD) if they present with slow performance in reaching at least two of the following milestones: gross or fine motor activity, speech or language, cognition or mental activity, and social or personal activities of daily living [1,2]. Individuals with congenital malformations (CM) had a problem in the heart, kidney, brain, muscles, or skeleton since birth, and individuals with intellectual disability (ID) had problems with general mental abilities: (i) intellectual functioning (such as learning, reasoning, problem-solving) and/or (ii) adaptive functioning (such as language, number concept, time calculation, memory, social responsibility, communication, and independent living) [3,4]. The Saudi Population Registry (statistics authority) reported a combined disabilities population of around 7.1%, including hearing impairment (n = 289,355), attention-deficit/hyperactivity disorder (n = 30,155), mobility impairment (n = 833,136), visual impairment (n = 811,610), autism spectrum disorder (n = 53,282), Down’s Syndrome (n = 19,428), etc., but nothing specific to DD/CM/ID. Genetic disorders and congenital abnormalities occur in 2–5% of all live births, causing approximately 50% of childhood deaths globally and approximately 35% of perinatal deaths in Saudi Arabia [5]. Until recently, conventional karyotyping was the method of choice for diagnosing individuals with DD/CM/ID in government-funded hospitals and clinics, but its diagnostic rate has been only ~5% because of major limitations such as size, accuracy, and specificity [6,7]. Although fluorescence in situ hybridization (FISH) has improved diagnostic yield by another 2–3%, identification of disease-associated chromosomal alterations or any well-known syndrome remains challenging. 

Genomic variations include single nucleotide variants (SNVs), small insertions or deletions (indels), copy number variations (CNVs), large structural variants (SVs), and abnormalities in chromosome number [8]. CNVs are genomic regions frequently gained or lost in a population, and the average individual harbors hundreds of CNVs [9]. Most are benign but some play a role in human disease through dosage imbalance, altered gene expression, and disruption of regulatory elements. Although CNVs are common in the human genome, they are rarely linked to genetic diseases [10]. Those linked to such diseases are associated with complex phenotypes and disease susceptibility because they alter gene copy numbers and gene expression. To analyze CNVs, the aforementioned low-resolution cytogenetic technologies such as karyotyping (approximately >10 Mbp) and FISH (5–10 Mbp) are routinely used [11], but they suffer from bottlenecks of low diagnostic rates and inability to detect short CNVs [12]. However, with the advent of array CGH and next-generation sequencing technology, one can now identify small variants of 10–25 kb using the former and even up to 50 bp using the latter [13,14,15]. 

Array CGH can help scan the entire genome at higher resolution and detect chromosomal alterations by comparing hybridization intensity between the DNA of a patient and a healthy control [16]. Two meta-analyses involving 46,298 and 28,526 individuals with genetic disorders have shown the clinical impact of array CGH, detecting 35% and 46% of pathogenic abnormalities, respectively [17,18]. Both the International Collaboration for Clinical Genomics and American College of Medical Genetics and Genomics (ACMG) have recommended array CGH as the first-tier cytogenetic diagnostic test for patients with DD, CM, and ID [19,20]. However, it has not been implemented in Saudi Arabia yet because of a lack of sufficient validation studies. 

Precise identification and accurate clinical annotation of CNVs are vital for evaluating patients with DD, CM, and ID. Recent guidelines issued jointly by ACMG and the Clinical Genome Resource (ClinGen) recommend classifying CNVs based on their pathogenicity [21]. Studies conducted to identify disease-associated CNVs have broadly classified them as follows: (i) pathogenic, causing common or rare syndromes, and (ii) variants of uncertain significance (VUSs), not known to be directly causative of a disease but sometimes associated with disease/disease-like conditions [6,7,22,23]. G-banding karyotyping is routinely used to detect chromosomal abnormalities in unexplained genetic diseases. Recently, however, array CGH has emerged as a high-resolution genetic screening method and may soon replace conventional karyotyping. Unfortunately, because array CGH is more expensive, it remains unavailable in most clinical laboratories, and countries like Saudi Arabia still mostly use standard karyotyping as the method of choice. 

The diagnostic application of array-CGH in Saudi DD/CM/ID patients, to the best of our knowledge, has not been reported yet. However, a few studies have reported the application of array-CGH in the identification of disease-causing variants such as recurrent spontaneous abortion in Saudi Arabia [24], juvenile myoclonic epilepsy [25], acute myeloid leukemia [26], Lynch Syndrome [27], gastric cancer [28], Williams’ syndrome [29], DiGeorge Syndrome [30], and congenital heart disease [31]. Hence, in the present study, we aimed to investigate the genetic heterogeneity in Saudi children with DD/CM/ID. We then discuss the characteristic features and clinical significance of the detected CNVs including pathogenic and VUS and compare the diagnostic yields of array CGH with previous reports.

## 2. Materials and Methods

### 2.1. Patients and Ethical Approval

We recruited 63 children with DD, CM, and/or ID after obtaining their informed consent from parents/guardians and research approval from the institutional ethics committee (approval Code # 012-CEGMR-ETH-0), and work was performed in accordance with the Declaration of Helsinki. Children below the age of 18 years who were diagnosed with distinct features of DD, CM, and ID and were residents of the Western region of Saudi Arabia were included in the study. Patients who refused to give informed consent were excluded. Clinical examination of the patients was conducted at the Center of Excellence in Genomic Medical Research referred by the KAU Hospital (Jeddah), the Maternity and Child Hospital (Jeddah), and the Pediatrics Clinic of Taif Hospital (Taif), all in Saudi Arabia, and were referred to the Center of Excellence in Genomic Medical Research for molecular cytogenetic testing. Clinical information and family history were recorded to establish the DD etiology and to elucidate the diagnostic process of unexplained DD/CM/ID.

### 2.2. Cytogenetics Analyses

Karyotyping based on G banding using Trypsin and Giemsa (GTG banding) was performed based on microscopic examination of at least 20 metaphases per case. Chromosomes were analyzed using Applied Imaging Karyotyping software (Applied Imaging, Santa Clara, CA, USA), and karyotypes were described according to the International System for Human Cytogenomic Nomenclature (ISCN, 2020) [24,32,33]. 

### 2.3. DNA Preparation and Whole-Genome Array CGH

Genomic DNA from 5 mL patient’s blood was extracted using QIAamp DNA Blood Mini Kit (Qiagen, Hilden, Germany) and purified using QIA-Miniprep Kit (Qiagen). The concentration and quality of DNA were determined using a NanoDrop 2000 spectrophotometer (Thermo Fisher Scientific, Waltham, MA, USA).

To investigate genome defects, we applied high-density array CGH using SurePrint G3 Human CGH Microarray Kit in 1 × 244 K (AMADID Number: 014693) and 2 × 400 K (AMADID Number: 021850) formats, consisting of 244,000 and 400,000 copy number probes, respectively (Agilent Technologies, Santa Clara, CA, USA), with UCSC hg18 as the reference genome. The overall median probe spacing of the 1 × 244 K and 2 × 400 K chip was 8.9 kb and 5.3 kb, respectively, whereas the spacing of RefSeq genes was 7.4 kb and 4.6 kb, respectively. Microarray analysis was conducted according to Agilent’s assay procedures, with modifications. Commercial human reference DNA was used (Agilent Technologies, Santa Clara, CA, USA). Upon being enzymatically digested using *Alu*I and *Rsa*I, the DNA samples were labeled with cyanine 3-deoxyuridine triphosphate (Cy3-dUTP) using SureTag DNA Labeling Kit (Agilent Technologies), whereas sex-matched reference DNA samples were labeled with Cy5-dUTP. The labeled DNA was purified before being mixed with Cot-1 DNA, 10× array CGH blocking agent, and 2× HI-RPM hybridization buffer (Agilent Technologies); this mixture was dispensed into a microarray slide. Hybridization was performed in an Agilent hybridization chamber at 67 °C and 20 rpm for 24 h and then washed stringently with wash buffer 1 and wash buffer 2 (Agilent Technologies). Microarray slide images were captured using Agilent SureScan Microarray Scanner G2505C.

### 2.4. Interpretation of CNVs

CNV analysis was performed using Agilent Cytogenomics v5.2.0.2 and human genome build hg18. A CNV was considered either a gain or loss if the region had at least three consecutive probes with a mean log_2_ ratio of ±0.25, respectively. A mean log_2_ ratio > 0.58 was considered a gain, whereas that <−1 indicated a loss. Following the recommended guidelines for detecting pathogenic variants, CNVs < 300 kb were excluded from further analysis. In addition, the CNVs were classified as benign if the corresponding regions did not harbor genes or were present in the healthy normal controls (Database of Genomic Variants, DGV; http://projects.tcag.ca/variation, accessed on 25 November 2022). 

To interpret and confirm the clinical significance of the CNVs, they were analyzed across multiple public databases, including UCSC (http://genome.ucsc.edu, accessed on 25 November 2022), DGV (http://dgv.tcag.ca/dgv/app/, accessed on 25 November 2022), OMIM (http://www.omim.org/, accessed on 26 November 2022), DECIPHER (http://decipher.sanger.ac.uk/, accessed on 26 November 2022), and PubMed (https://www.ncbi.nlm.nih.gov/pubmed/, accessed on 27 November 2022). Pathogenic CNVs (common and rare syndromic/non-syndromic) were identified for DD/CM if they overlapped with previously reported pathogenic CNVs. Novel VUSs was identified by exploring genomic alterations, including microdeletions/duplications. The detected CNVs were classified as VUS if any genes present in this region were linked to known functions but could not be directly associated with the disease under investigation.

### 2.5. Quantitative Real-Time PCR

Quantitative real-time PCR (qPCR) was used to validate the deletions and duplications of CNVs detected by array-CGH. The primer sets were designed for selected genomic regions of the target genes including *FLI1*, *SHANK3*, and *MBP*, and an endogenous GAPDH gene as an internal control using Primer-3 Software (V.0.4.0). The reaction was run in a final volume of 10 μL, comprising of 5 μL SYBR-Green qPCR master mix (KAPA Biosystems, Wilmington, NC, USA), 10 pmol of each primer, and 20 ng genomic DNA. The PCR was performed in triplicate using SYBR-Green qPCR master mix (KAPA Biosystems, USA) in a 96-well plate. Raw data was generated by StepOne Plus™ Real-Time PCR Systems and Data Assist software. qPCR data were analyzed by ∆∆C_T_ or Livak method and the Graph Pad PRISM software was used for presentation.

## 3. Results

### 3.1. Clinical Finding

We examined 63 DD/CM/ID patients for genome defects using chromosomal microarray analysis. They exhibited complex DD/CM/ID with distinct additional features, including delayed speech (n = 20), congenital heart defects (n = 12), dysmorphic features (n = 15), microcephaly (n = 7), hypotonia (n = 4) and ID (n = 5). The patient male-to-female ratio was 1.42, and the mean age was 2.3 years (ranging from 8 days to 16 years). Most individuals were children aged 1–5 years (n = 34), followed by those aged 6–15 years (n = 18), <1 year (n = 9) and >15 years (n = 2) (Supplementary Table S1). 

### 3.2. Cytogenetic Abnormalities

According to the GTG banding karyotype analysis, ten individuals had chromosomal abnormalities, including Turner syndrome, Klinefelter syndrome, Edward syndrome, and gain/loss within chromosomes 1, 9, 11, 13, 14, 18, and 22 (Table 1). These abnormalities were confirmed by the array CGH results. Notably, an 8-day-old patient [BL-401-13] with DD, congenital heart disease, and dysmorphic features exhibited two types of chromosomal abnormalities: translocation t(13;14)(q10;q10) and trisomy 18.

### 3.3. Pathogenic CNVs

Array CGH detected chromosomal abnormalities in 28% of the patients (n = 19; 10 male and 8 female), whereas conventional G-band karyotyping detected chromosomal abnormalities in 15.87%. Two-thirds of the patients with pathogenic CNVs were under 5 years of age, including 3 newborns (0–2 months), 9 infants (2–12 months), 22 toddlers (>1–4 years), and 9 children (>4–5 years). 

### 3.4. Characteristics of Disease-Associated CNVs

**Figure 1:**
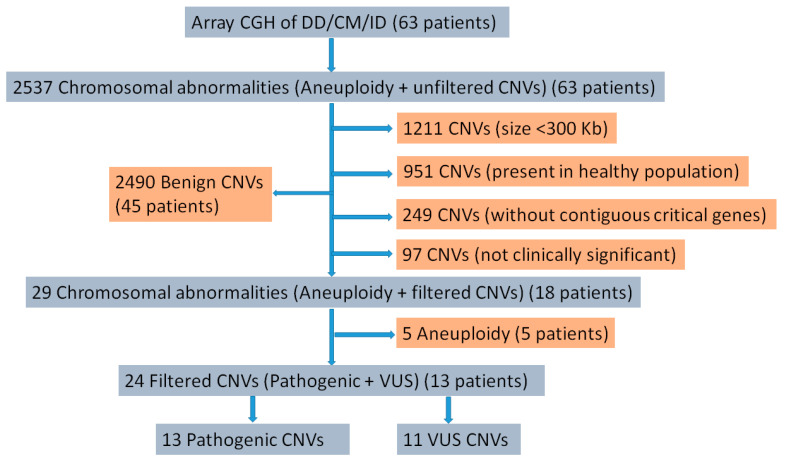
Flowchart for the filtration of pathogenic/VUS CNVs in Whole-Genome Array CGH results. Out of 2537 chromosomal abnormalities, 5 aneuploidy, 1211 CNVs of <300 Kb, 951 CNVs present in a healthy population, 249 CNVs without contiguous critical genes, and 97 non-clinically significant CNVs were removed to filter out 24 pathogenic/VUS CNVs among 13 DD/CM/ID patients.

Investigations with two array-CGH chips (1 × 244 K, n = 11; 2 × 400 K, n = 52) revealed 2537 CNVs, including 1326 CNVs of ≥300 kb and 1211 CNVs of <300 kb. However, only 24 CNVs (from 13 individuals) qualified as causative for disease (Table 2). Additionally, five individuals were detected with chromosomal aneuploidy (two 47,XXY, two 45,X, and one a patient with trisomy 18 on a karyotype carrying a balanced translocation between chromosome 13 and 14 (46,XX,t(13;14)(q10;q10)+18) were excluded from CNV analysis. The remaining 2490 CNVs were classified as benign if they were present in the healthy population, <300 kb in size, without any contiguous critical genes, or not clinically significant (Figure 1). The prevalence of chromosomal loss (58%, 14/24) was greater than that of chromosomal gain (42%, 10/24). Gains/duplications were found in 8q24, 9p24p13, 11q12q13, and 18p11 CNVs, while losses/deletions were found in 3p23p14, 10q26, 11p15, 11q24q25, 13q21.1q32.1, 15q25q26, 16p13.3p11.2, and 20q11.1q13.2. Interestingly, both gain and loss were found in some CNVs, including 16q21q23, 18p11.32p11.21,and 22q11q13 in different individuals.

Based on their association with disease phenotypes and prevalence in previous studies and the OMIM database, the clinically significant CNVs were classified into two groups, specifically pathogenic (52%, 13/24 CNVs) and VUSs with variable effects (48%, 11/24 CNVs). Pathogenic CNVs were 8q24, 9p24p13, 10q26, 11p15, 22q11q13, 11q24q25, 15q25q26, and 16p13p11, and 18p11,. VUS CNVs were 3p23p14, 11q12.1q14, 13q21.1q32.1, 16q21q23, 18q23, 20q11.1q13.2, and 22q11q13. Interestingly, 22q11q13 was detected in at least four individuals, while 16q21q23.1 and 18p11.32p11.21 were found in three individuals. 

We discovered that in a few cases, the size of the chromosomal abnormalities overlapped, including contiguous essential genes for the same syndrome. Sizes of the disease-associated CNVs varied and can be grouped as: <5 Mb (17.8%), 5–10 Mb (13.5%), 10–20 Mb (22.7%), 20–25 Mb (18.4%), and >25 Mb (27.6%). The total number of detected CNVs was 24 while distinct non-overlapping CNVs were 16. The 3p23p14.2 deletion was detected in one patient [BL-363-12]. 3p23p14 deletion (Robinow syndrome 1, Septo-optic dysplasia, Spondylocarpotarsal synostosis syndrome) causes severe intellectual disability, abnormal physical features, and developmental delay in language and motor skills. 

**Figure 2:**
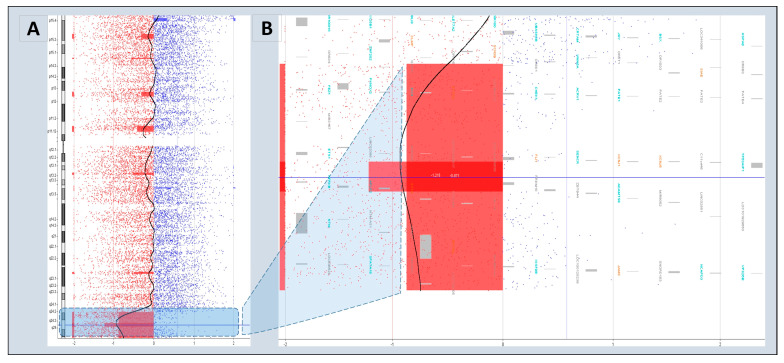
(**A**) Chromosomal microarray analysis showing 10.8 Mb subtelomeric deletions of chromosome 11q24.2q25 in a 4-year-old male [BL-080-12] patient with developmental delay, delayed speech, and dysmorphic features. (**B**) Detected region of chr11: 123,615,752-134,432,324bp encompasses many pathogenic genes, including *FLI1*, *KCNJ1*, *KCNJ5*, *ETS1*, and *JAM3* that cause Jacobsen syndrome.

We detected a rare microdeletion of the 8q24.3 region in a 6-year-old patient [BL-1086-11] with intellectual disability, ADHD, dysmorphic, seizures, small ears, triangular Face, VSD at birth, no speech, delayed walking, and epilepsy. A gain of 38.5 Mb at 9p24.3p13.1 was detected in a 2.6 year old girl [BL-181-13] with hypotonia, delayed speech, not walking, cleft-lip, VSD, ASD, pulmonary stenosis, and dysmorphic features. A rare 10q26.13-q26.3 deletion was detected in a female of 1.6 years [BL-464-12] with short stature and DD. Furthermore, we detected three CNVs in chromosome 11, including deletion of 11p15.5p15.4 [BL-1086-11], duplication of 11q12.11q13.3 [BL-210-12], and deletion of 11q24.2q25 [BL-080-12] (Figure 2). 

**Figure 3:**
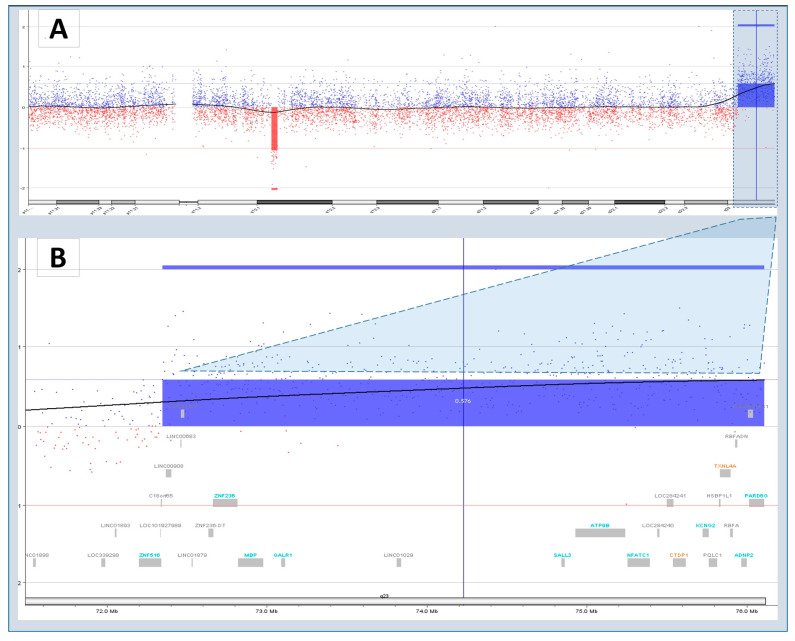
(**A**) Chromosomal microarray analysis showing 3.7 Mb duplication of 18q23 in a 2-year-old patient [BL-464-12] with developmental delay and short stature. (**B**) Detected region (chr18: 72346539_76111023bp) encompasses many pathogenic genes including *MBP*, *CTDP*, *GALR1*, and *TXNL4A* genes responsible for demyelination, failure of growth hormone stimulation response, congenital cataracts, facial dysmorphism, neuropathy, and sensorineural deafness.

**Figure 4:**
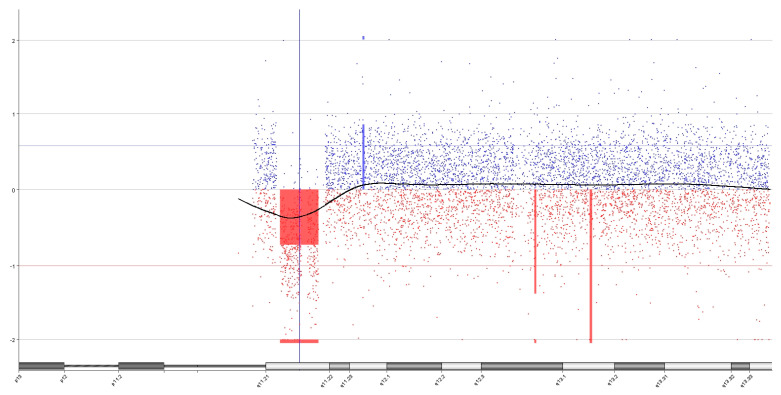
Chromosomal microarray analysis showing 2.5 Mb deletions of 22q11.21 in a three-month-old patient [BL-363-12] with DD, congenital heart disease, and DiGeorge syndrome.

A 4-month-old [BL-461-12] with DD, dysmorphic features, short stature, failure to thrive, small finger with two phalanges, and a simian crease in left hand harbored a 13q21.1q32.1 deletion. Deletion of the chromosome 15 q arm (15q13.1q26.1, 62.6 Mb) was detected in a 2-year-old [BL-363-12] with DD, speech delay, and dysmorphic features. A deletion and duplication of the chromosome 16 p arm (16p13.3p11.2, 32.6 Mb) was detected in a 2-year-old [BL-363-12] with DD, speech delay, and dysmorphic features, and in a 6-year-old [BL-210-12] with DD and delayed speech, respectively. Another deletion and duplication of 16q21q23.1 were detected in a 1-year-old [BL-161-14] with DD, VSD, and failure to thrive, and a 2-year-old [BL-210-12] with DD, delayed speech, and dysmorphic feature, respectively. A 1-month-old [BL-597-12] with dysmorphic features, low-set ears, and closed VSD and a 16-year-old [BL-902-10] with DD, microcephaly, ID, and dysmorphic features had 18p11.32-p11.21 duplication. Duplication of 18q23 was found in a 1.5-year-old [BL-464-12] with DD and short stature (Figure 3). A deletion of 20q11.1q13.2 was found in a 2-year-old [BL-363-12] with DD, speech delay, and dysmorphic features. A duplication of 22q11.1-q13.33 was found in a 6-year-old [BL-210-12] with DD and speech delay; a deletion of 22q11.21 was present in a 3-month-old [BL-793-13] with DD, congenital heart disease, and DiGeorge syndrome; and a deletion 22q11.1q13.31 was identified in a 2-year-old [BL-363-12] with DD, speech delay, and dysmorphic features (Figure 4). 

### 3.5. Confirmation of CNVs by Quantitative Real-Time PCR (qPCR) 

**Figure 5:**
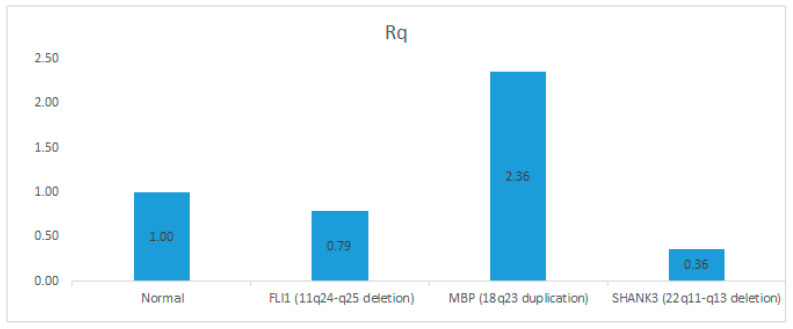
Confirmation of CNVs by qPCR: Bar graph showing a significant decrease in copy number of *FLI1* and *SHANK3*, and a significant increase in *MBP*.

qPCR results confirmed the CNVs detected by array CGH. We found a significant decrease in the gene copy number of *FLI1* (11q24q25 deletion) and *SHANK3* (22q11q13 deletion), and a significant increase in *MBP* (18q23 duplication) (Figure 5).

## 4. Discussion

In this study, we identified the CNVs associated with DD/CM/ID, and the diagnostic yield of array CGH (28%) was in accordance with previous findings [1,19,34]. The clinical significance of identified CNVs was classified into three categories: established clinical significance (syndromic pathogenic and non-syndromic pathogenic), VUSs, and without clinical significance (benign). The pathogenic CNV is directly associated with patient phenotype, the non-syndromic CNV encompasses diverse disorders that are indirectly associated with individual phenotype, and the VUS is linked to indistinct disease vulnerability. Rare chromosomal abnormalities with no systematic analysis of limited cases, where common clinical manifestations remain elusive, were also classified as VUSs. 

The human genome is diploid and expected to contain two copies of each autosome, except the sex chromosome in men. However, in reality, genetic variations are commonly present, ranging from large chromosome anomalies and copy number variations to single nucleotide changes in the human genome. CNVs, usually DNA segment size range from 1 Kb to 10 Mb, are present at variable copy numbers in comparison with a reference genome. Chromosome abnormalities are less frequent, but their presence leads to different diseases/syndromes, while CNVs and SNPs are frequently present in the genome as benign but a few are pathogenic as well [35,36]. In general, humans harbor 10–100 CNVs, which are mostly benign [37]. Herein, we detected 2537 unfiltered CNVs among 63 individuals (~40 CNVs per person) with most of them being benign. Only 42 CNVs across 19 patients (~2 CNVs per person) were pathogenic or VUSs. 

Microdeletions in 8q24.3 specifically *PUF60* are linked to the rare Verheij syndrome [38]. Duplication of the 9p24.3p13.1 region involving the *SMARCA2* gene cause Nicolaides–Baraitser syndrome, which may be associated with DD, ID, microcephaly, and short stature [39]. 

A deletion of 10q26.13q26.3 region (8 Mb) containing the *DOCK1* and *C10ORF90* genes was an extremely rare chromosomal abnormality with less than 100 reported cases of DD, ID, dysmorphic facial features, and heart problems as well as skeletal and urogenital abnormalities [40]. The deletion of 11p15.5, including the *CDKN1C* and *KCNQ1OT1* genes, is associated with Beckwith–Wiedemann syndrome [41]. A deletion of the 11q12.2q14.1 region encompassing (*DAGLA, BEST1*, *SPTBN2*, *SHANK2*, *FADD*, *FGF3*, *KMT5B*, *GAL*, *PHOX2A* and *CLPB*) genes are known to cause spinocerebellar ataxia, deafness, epilepsy, fibrosis of muscle type 2 and 3-methylglutaconic aciduria type VII [42,43]. Additionally, a deletion of 11q24.2q25, associated with Jacobsen syndrome, is a rare chromosomal disorder (1 in 100,000) manifesting as growth and psychomotor retardation [44,45]. 

A deletion of 15q13.1-q26.1, including *HERC2*, *STRC*, *CHRNA7*, *FBN1*, *CAPN3*, *DNAAF4*, *KBTBD13*, *SMAD3*, *CIB2*, and *OTUD7A* genes, was a rare 15q13.3 and 15q25 microdeletion syndrome with global DD, ID, hypotonia, and facial dysmorphism [46,47,48,49]. Deletion and duplication of the 16p13.3-p11.2 region includes 16p11.2 duplication syndrome and 16p13.3 deletion/duplication syndrome [50]. The 16q22 deletion syndrome is associated with DD, hypotonia, neurological disorders, failure to thrive, and dysmorphic features [51]. The deletion of 16q21q22 has also been reported with cleft soft palate and dysmorphic features [52]. 

A duplication of 18p11.32p11.21 and 18q11.1q23 has been reported with rare conditions of DD, short stature, ID, and dysmorphic facial features in two individuals [53]. A microduplication of 3.7 Mb at 18q23 encompassing the *CTDP* and *TXNL4A* genes are reported to be associated with a failure to respond to growth hormone stimulation, facial dysmorphism, neuropathy, and sensorineural deafness [54]. A 20q11.1q13.2 deletion encompassing *CHMP4B*, *SEC23B*, and *OVOL2* genes are linked to DD, ID, skeletal abnormalities, and heart defects [55,56,57]. A 22q11.21 microdeletion encompassing the *TBX1, SHANK3*, *SOX10*, *AP1B1*, *FBXO7*, *MYH9*, and *SPECC1L* genes cause DiGeorge syndrome, which is associated with DD, ID, seizure, and cardiac malformations, with a prevalence of 1 in 4000 [58,59]. A 22q11 deletion also causes velocardiofacial syndrome, conotruncal anomaly face syndrome, and tetralogy of fallot [60]. 

Our data along with previous research demonstrates that array CGH is efficient in identifying known and novel disease associated CNVs. Extensive CNV analysis of developmental disabilities identified eight VUSs (3p23p14, 11q12.1q14, 13q21.1q32.1, 16p13p11, 16q21q23, 18q23, 20q11.1q13.2, and 22q11q13 [61]. 

There are some limitations of the study, but it still has future directions. The cohort size was not large enough to truly represent the population. Clinical pictures and images were not available for publication because of confidentiality and the patient’s privacy policy. The discrepancy was found in the G-banding and the array CGH result because a couple of big CNVs (>10 Mb) were detected by array CGH but not found in G-banding, despite using the allowed resolution band of 550, and this might be because the traditional technique lacked accuracy. The array CGH confirmed all karyotyping results, but was limited in detecting balanced translocations or inversions, ring chromosomes, and low-level mosaicism. An additional challenge lies in interpreting VUSs found through array CGH and validating their clinical significance. These considerations lead us to recommend that diagnoses employ both array CGH and conventional karyotyping to confirm positive cases and identify CNVs in negative cases. In the future, the validation of detected CNVs, especially VUSs, and their confirmation on a bigger cohort will overcome the limitations of the current study.

## 5. Conclusions

This is a first array CGH-based comprehensive study from Saudi Arabia, and for the first time we herein, we report the extremely rare pathogenic CNVs/genes (8q24, 10q26, 11q24q25, 18p11, 15q25q26, and 16p13p11) among Saudi individuals with DD, CM, and ID that may contribute to their genetic etiology. Additionally, our result showed a couple of potential causative CNVs that may be re-classified as pathogenic CNVs after detailed validation and functional characterization. Our results enhanced the knowledge of the copy number variants underlying DD, CM, and ID in the Saudi population, and array technology will potentially help to improve the genetic diagnosis of CNVs and novel syndromes in neonatal and prenatal cases.

## Figures and Tables

**Table 1:** Chromosomal abnormalities detected using G-banding and array comparative genomic hybridization in pediatric developmental delay/congenital malformations/intellectual disability patients.

S. No	Biobank Code	G-Banding Result	Array CGH Results
1	BL-080-12	46,XY,del(11)(q24)	arr[hg18] 11q24.2q25(123615752_134432324)x1
2	BL-181-13	47,XX,+del(9)(q22)	arr[hg18] 9p24.3p13.1(194193_38745183)x3
3	BL-210-12	46,XX,dup(3)p36-p33	arr[hg18]1p36.33p33(746956_48281628)x3
4	BL-401-13	46,XX,t(13;14)(q10;q10) + 18 *	arr[hg18] 18p11.32p11.21 (75432_14092527)x3 arr[hg18] 18q11.1q23(16910049_76111023)x3
5	Bl-628-12	45,X	arr[hg18]Xp22.33p11.1(2711273_58499110)x1 arr[hg18]Xq11.1q28(61848414_154582526)x1
6	BL-664-11	47,XXY	arr[hg18]Xp22.33p11.1(2711273_58499110)x4 arr[hg18]Xq11.1q28(61848414_154570236)x4
7	BL-664-13	45,X	arr[hg18] Xp22.33p11.1(2711273_58499110)x1 arr[hg18] Xq11.1q28(61848414_154582526)x1
8	BL-793-13	46,XY,del(22)(q11.2q11.2)	arr[hg18] 22q11q21(17269039_19794119)x1
9	BL-902-10	47,XY,+mar or 47,XY,+i(18)(p10)	arr[hg18] 18p11.32p11.21(114641_15062794)x4
10	BL-1288-13	47,XXY	arr[hg18] Xp22.33p11.1(2711273_58499110)x4 arr[hg18] Xq11.1q28(61848414_154570236)x4

* The balanced translocation between chromosomes 13 and 14 was not identified by aCGH.

**Table 2:** Pathogenic and variants of uncertain significance and syndromic copy number variations detected by array CGH in pediatric developmental delay, congenital malformations, and intellectual disability patients.

S. No	Biobank Code	CNVs	CNV Classification	OMIM Genes	Associated Syndromes
1	BL-080-12	arr[hg18]11q24.2q25(123615752_134432324)x1	Pathogenic	*FLI-1*, *KCNJ1*, *KCNJ5*, *JAM3*	Jacobsen syndrome
2	BL-161-14	arr[hg18]16q21q23.1(65021970_74573408)x1	VUS	*CDH11*, *CBFB*, *TK2*, *GOT2*, *BEAN,*	16q22 deletion syndrome
3	BL-181-13	arr[hg18]9p24.3p13.1(194193_38745183)x3	Pathogenic	*SMARCA2*, *VLDLR*	Nicolaides-Baraitser syndrome
4	BL-210-12	arr[hg18]11q12.2- q13.5(60243525_ 75206053)x3	VUS	*DAGLA*, *FADD*, *FGF3*	Spinocerebellar ataxia 20
		arr[hg18]16p13.3 p11.2(28087_ 31446768)x3	Pathogenic	*CREBBP*, *DNASE*, *TRAP1*, *TELO2*	16p13.3 duplication syndrome
		arr[hg18]22q11.1q13.33(15834835_ 49525130)x3	VUS	*LARGE*, *SHANK3*, *TBX1*, *HPS4*	Duplication of 22q12/13
5	BL-363-12	arr[hg18]3p23p14.2(31581603_58552982)x1	VUS	*WNT5A*, *HESX1*, *FLNB*, *CCK*, *GLYCTK*	Robinow syndrome 1
		arr[hg18]15q13.1q26.1(27196809_89833172)x1	Pathogenic	*CHRNA7 and OTUD7*	15q13.3 microdeletion syndrome
		arr[hg18]16p13.3p11.2(1103363_33495560)x1	VUS	*CREBBP*, *DNASE*, *TRAP1*, *TELO2*	16p13.3 deletion syndrome
		arr[hg18]20q11.1q13.2(28252372_50274994)x1	VUS	*GDF5*, *EPB41L1*, *SAMHD1*	20q deletion
		arr[hg18]22q11.1q13.31(14513474_44350783)x1	VUS	*PEX26*, *SHANK3*, *TBX1*, *HPS4*	Deletion of 22q12/13
6	BL-457-12	arr[hg18]16q21q23.1(65021970_74573408)x1	VUS	*CDH11*, *CBFB*, *TK2*, *GOT2*, *BEAN*, *KARS1*	16q22 deletion syndrome
7	BL-461-12	arr[hg18]13q21.1- q32.1(57009987_95317681)x1	VUS	*DIAPH3*, *PIBF1*, *TBC1D1*, *FBXL3*, *EDNRB*, *PCDH17*	DD, ID, skeletal and other abnormalities
		arr[hg18]16q21-q23.1(65109284_74852983)x3	VUS	*CDH11*, *CBFB*, *TK2*, *GOT2*, *BEAN*, *KARS1*	16q22 duplication syndrome
8	BL-464-12	arr[hg18]10q26.13q26.3(127272748_135284168)x3	Pathogenic	*DOCK1*, *C10ORF90*	10q26 duplication syndrome
		arr[hg18]18q23(7234653976111023)x1	VUS	*CTDP1*, *GALR1*	18q23 deletion syndrome
9	BL-518-13	arr[hg18] 15q25.1q26.3(78427008_100298411)x3	Pathogenic	*AP3B2*, *HOMER2*, *SH3GL3*, *CHD2*	Levy-Shanske syndrome
10	BL-597-12	arr[hg18]18p11.32p11.21(121700_14112521)x4	Pathogenic	*TGIF1*, *NDUFV2*, *PIEZO2*, *GNAL*	Partial trisomy 18p
		arr[hg18]22q11.1q13.33(15681796_49571118)x3	VUS	*LARGE*, *SHANK3*, *TBX1*, *HPS4*	Duplication of 22q12/13
11	BL-793-13	arr[hg18]22q11.21(17269039_19794119)x1	Pathogenic	*PRODH*, *SLC25A1*, *SNAP29*, *TBX1*	DiGeorge Syndrome
12	BL-902-12	arr[hg18]18p11.32p11.21(114641_15062794)x4	Pathogenic	*TGIF1*, *NDUFV2*, *PIEZO2*, *GNAL*	Partial trisomy 18p
13	BL-1086-11	arr[hg18]8q24.3(142081172_146142017)x1	Pathogenic	*PUF60*, *GRINA*	Verheij syndrome
		arr[hg18]11p15.5p15.4(234177_2917590)x1	Syndromic	*CDKN1C*, *IGF2*, *KCNQ1OT1*	Beckwith-Wiedemann syndrome

## Data Availability

The raw datasets used in this study are available at GEO repository with accession number of GSE182101 (super series), GSE181995 (subseries) and GSE182081 (subseries).

## Supplementary Materials

The following supporting information can be downloaded at: https://www.mdpi.com/article/10.3390/children10040662/s1, Table S1: Clinical information of pediatric developmental delay, congenital malfunction, and intellectual disability patients included in this study.

## Abbreviations

DD: Developmental delay; ID: Intellectual Disability; CM: Congenital malformations; Array CGH: Array comparative genomic hybridization; CNVs: Copy number variations; VUS: Variable of unknown significance; ID: Intellectual disability; ASD: Atrial septal defect; VSD: Ventricular septal defect; WBS: William Beuren syndrome; ADHD: Attention deficit hyperactivity disorder; OMIM: Online Mendelian Inheritance in Man.

## References

[1] Mithyantha R., Kneen R., McCann E., Gladstone M. (2017). Current evidence-based recommendations on investigating children with global developmental delay. Arch. Dis. Child..

[2] Shevell M., Ashwal S., Donley D., Flint J., Gingold M., Hirtz D., Majnemer A., Noetzel M., Sheth R.D. (2003). Practice parameter: Evaluation of the child with global developmental delay: Report of the Quality Standards Subcommittee of the American Academy of Neurology and The Practice Committee of the Child Neurology Society. Neurology.

[3] Jones K.L., Adam M.P. (2015). Evaluation and diagnosis of the dysmorphic infant. Clin. Perinatol..

[4] Patel D.R., Cabral M.D., Ho A., Merrick J. (2020). A clinical primer on intellectual disability. Transl. Pediatr..

[5] Fida N.M., Al-Aama J., Nichols W., Nichols W., Alqahtani M. (2007). A prospective study of congenital malformations among live born neonates at a University Hospital in Western Saudi Arabia. Saudi Med. J..

[6] Hussein I.R., Bader R.S., Chaudhary A.G., Bassiouni R., Alquaiti M., Ashgan F., Schulten H.-J., Al Qahtani M.H. (2018). Identification of De Novo and Rare Inherited Copy Number Variants in Children with Syndromic Congenital Heart Defects. Pediatr. Cardiol..

[7] Lee C.L., Lee C.H., Chuang C.K., Chiu H.C., Chen Y.J., Chou C.L., Wu P.S., Chen C.P., Lin H.Y., Lin S.P. (2019). Array-CGH increased the diagnostic rate of developmental delay or intellectual disability in Taiwan. Pediatr. Neonatol..

[8] Alkan C., Coe B.P., Eichler E.E. (2011). Genome structural variation discovery and genotyping. Nat. Rev. Genet..

[9] Conrad D.F., Pinto D., Redon R., Feuk L., Gokcumen O., Zhang Y., Aerts J., Andrews T.D., Barnes C., Campbell P. (2010). Origins and functional impact of copy number variation in the human genome. Nature.

[10] Redon R., Ishikawa S., Fitch K.R., Feuk L., Perry G.H., Andrews T.D., Fiegler H., Shapero M.H., Carson A.R., Chen W. (2006). Global variation in copy number in the human genome. Nature.

[11] Buysse K., Delle Chiaie B., Van Coster R., Loeys B., De Paepe A., Mortier G., Speleman F., Menten B. (2009). Challenges for CNV interpretation in clinical molecular karyotyping: Lessons learned from a 1001 sample experience. Eur. J. Med. Genet..

[12] Duan J., Zhang J.-G., Deng H.-W., Wang Y.-P. (2013). Comparative studies of copy number variation detection methods for next-generation sequencing technologies. PLoS ONE.

[13] Carter N.P. (2007). Methods and strategies for analyzing copy number variation using DNA microarrays. Nat. Genet..

[14] Moreno-Cabrera J.M., Del Valle J., Castellanos E., Feliubadaló L., Pineda M., Brunet J., Serra E., Capellà G., Lázaro C., Gel B. (2020). Evaluation of CNV detection tools for NGS panel data in genetic diagnostics. Eur. J. Hum. Genet. EJHG.

[15] Zhang L., Bai W., Yuan N., Du Z. (2019). Comprehensively benchmarking applications for detecting copy number variation. PLoS Comput. Biol..

[16] Bi W., Borgan C., Pursley A.N., Hixson P., Shaw C.A., Bacino C.A., Lalani S.R., Patel A., Stankiewicz P., Lupski J.R. (2013). Comparison of chromosome analysis and chromosomal microarray analysis: What is the value of chromosome analysis in today’s genomic array era?. Genet. Med..

[17] Ellison J.W., Ravnan J.B., Rosenfeld J.A., Morton S.A., Neill N.J., Williams M.S., Lewis J., Torchia B.S., Walker C., Traylor R.N. (2012). Clinical utility of chromosomal microarray analysis. Pediatrics.

[18] Riggs E.R., Wain K.E., Riethmaier D., Smith-Packard B., Faucett W.A., Hoppman N., Thorland E.C., Patel V.C., Miller D.T. (2014). Chromosomal microarray impacts clinical management. Clin. Genet..

[19] Miller D.T., Adam M.P., Aradhya S., Biesecker L.G., Brothman A.R., Carter N.P., Church D.M., Crolla J.A., Eichler E.E., Epstein C.J. (2010). Consensus statement: Chromosomal microarray is a first-tier clinical diagnostic test for individuals with developmental disabilities or congenital anomalies. Am. J. Hum. Genet..

[20] Syrmou A., Tzetis M., Fryssira H., Kosma K., Oikonomakis V., Giannikou K., Makrythanasis P., Kitsiou-Tzeli S., Kanavakis E. (2013). Array comparative genomic hybridization as a clinical diagnostic tool in syndromic and nonsyndromic congenital heart disease. Pediatr. Res..

[21] Riggs E.R., Andersen E.F., Cherry A.M., Kantarci S., Kearney H., Patel A., Raca G., Ritter D.I., South S.T., Thorland E.C. (2020). Technical standards for the interpretation and reporting of constitutional copy-number variants: A joint consensus recommendation of the American College of Medical Genetics and Genomics (ACMG) and the Clinical Genome Resource (ClinGen). Genet. Med. Off. J. Am. Coll. Med. Genet..

[22] Moeschler J.B., Shevell M. (2014). Comprehensive Evaluation of the Child With Intellectual Disability or Global Developmental Delays. Pediatrics.

[23] Serra-Juhé C., Rodríguez-Santiago B., Cuscó I., Vendrell T., Camats N., Torán N., Pérez-Jurado L.A. (2012). Contribution of Rare Copy Number Variants to Isolated Human Malformations. PLoS ONE.

[24] Karim S., Jamal H.S., Rouzi A., Ardawi M.S.M., Schulten H.J., Mirza Z., Alansari N.A., Al-Quaiti M.M., Abusamra H., Naseer M.I. (2017). Genomic answers for recurrent spontaneous abortion in Saudi Arabia: An array comparative genomic hybridization approach. Reprod. Biol..

[25] Naseer M.I., Rasool M., Chaudhary A.G., Sogaty S., Karim S., Schulten H.J., Bibi F., Pushparaj P.N., Algahtani H.A., Al-Qahtani M.H. (2017). Chromosomal Micro-aberration in a Saudi Family with Juvenile Myoclonic Epilepsy. CNS Neurol. Disord. Drug Targets.

[26] Ibrahim S.M., Karim S., Abusamra H., Pushparaj P.N., Khan J.A., Abuzenadah A.M., Gari M.A., Bakhashab S., Ahmed F., Al-Qahtani M.H. (2018). Genomic amplification of chromosome 7 in the Doxorubicin resistant K562 cell line. Bioinformation.

[27] Rasool M., Pushparaj P.N., Mirza Z., Imran Naseer M., Abusamra H., Alquaiti M., Shaabad M., Sibiany A.M.S., Gauthaman K., Al-Qahtani M.H. (2020). Array comparative genomic hybridization based identification of key genetic alterations at 2p21-p16.3 (MSH2, MSH6, EPCAM), 3p23-p14.2 (MLH1), 7p22.1 (PMS2) and 1p34.1-p33 (MUTYH) regions in hereditary non polyposis colorectal cancer (Lynch syndrome) in the Kingdom of Saudi Arabia. Saudi J. Biol. Sci..

[28] Bibi F., Ali I., Naseer M.I., Ali Mohamoud H.S., Yasir M., Alvi S.A., Jiman-Fatani A.A., Sawan A., Azhar E.I. (2018). Detection of genetic alterations in gastric cancer patients from Saudi Arabia using comparative genomic hybridization (CGH). PLoS ONE.

[29] Hussein I.R., Magbooli A., Huwait E., Chaudhary A., Bader R., Gari M., Ashgan F., Alquaiti M., Abuzenadah A., AlQahtani M. (2016). Genome wide array-CGH and qPCR analysis for the identification of genome defects in Williams’ syndrome patients in Saudi Arabia. Mol. Cytogenet..

[30] Bahamat A.A., Assidi M., Lary S.A., Almughamsi M.M., Peer Zada A.A., Chaudhary A., Abuzenadah A., Abu-Elmagd M., Al-Qahtani M. (2018). Use of Array Comparative Genomic Hybridization for the Diagnosis of DiGeorge Syndrome in Saudi Arabian Population. Cytogenet. Genome Res..

[31] Albesher N., Massadeh S., Hassan S.M., Alaamery M. (2022). Consanguinity and Congenital Heart Disease Susceptibility: Insights into Rare Genetic Variations in Saudi Arabia. Genes.

[32] Deans Z.C., Ahn J.W., Carreira I.M., Dequeker E., Henderson M., Lovrecic L., Õunap K., Tabiner M., Treacy R., van Asperen C.J. (2022). Recommendations for reporting results of diagnostic genomic testing. Eur. J. Hum. Genet..

[33] McGowan-Jordan J.O., Hastings R.J.O., Moore S.A., ISCN (2020). An International System for Human Cytogenomic Nomenclature (2020).

[34] Park S.J., Jung E.H., Ryu R.S., Kang H.W., Chung H.D., Kang H.Y. (2013). The clinical application of array CGH for the detection of chromosomal defects in 20,126 unselected newborns. Mol. Cytogenet..

[35] Erickson A., He M., Berglund E., Marklund M., Mirzazadeh R., Schultz N., Kvastad L., Andersson A., Bergenstråhle L., Bergenstråhle J. (2022). Spatially resolved clonal copy number alterations in benign and malignant tissue. Nature.

[36] Hovhannisyan G., Harutyunyan T., Aroutiounian R., Liehr T. (2019). DNA Copy Number Variations as Markers of Mutagenic Impact. Int. J. Mol. Sci..

[37] O’Byrne M.L., Yang W., Mercer-Rosa L., Parnell A.S., Oster M.E., Levenbrown Y., Tanel R.E., Goldmuntz E. (2014). 22q11.2 Deletion syndrome is associated with increased perioperative events and more complicated postoperative course in infants undergoing infant operative correction of truncus arteriosus communis or interrupted aortic arch. J. Thorac. Cardiovasc. Surg..

[38] Verheij J.B., de Munnik S.A., Dijkhuizen T., de Leeuw N., Olde Weghuis D., van den Hoek G.J., Rijlaarsdam R.S., Thomasse Y.E., Dikkers F.G., Marcelis C.L. (2009). An 8.35 Mb overlapping interstitial deletion of 8q24 in two patients with coloboma, congenital heart defect, limb abnormalities, psychomotor retardation and convulsions. Eur. J. Med. Genet..

[39] Guilherme R.S., Meloni V.A., Perez A.B.A., Pilla A.L., de Ramos M.A.P., Dantas A.G., Takeno S.S., Kulikowski L.D., Melaragno M.I. (2014). Duplication 9p and their implication to phenotype. BMC Med. Genet..

[40] Vera-Carbonell A., López-González V., Bafalliu J.A., Ballesta-Martínez M.J., Fernández A., Guillén-Navarro E., López-Expósito I. (2015). Clinical comparison of 10q26 overlapping deletions: Delineating the critical region for urogenital anomalies. Am. J. Med. Genet. Part A.

[41] Brioude F., Kalish J.M., Mussa A., Foster A.C., Bliek J., Ferrero G.B., Boonen S.E., Cole T., Baker R., Bertoletti M. (2018). Clinical and molecular diagnosis, screening and management of Beckwith–Wiedemann syndrome: An international consensus statement. Nat. Rev. Endocrinol..

[42] Stessman H.A., Xiong B., Coe B.P., Wang T., Hoekzema K., Fenckova M., Kvarnung M., Gerdts J., Trinh S., Cosemans N. (2017). Targeted sequencing identifies 91 neurodevelopmental-disorder risk genes with autism and developmental-disability biases. Nat. Genet..

[43] Knight M.A., Hernandez D., Diede S.J., Dauwerse H.G., Rafferty I., van de Leemput J., Forrest S.M., Gardner R.J., Storey E., van Ommen G.J. (2008). A duplication at chromosome 11q12.2-11q12.3 is associated with spinocerebellar ataxia type 20. Hum. Mol. Genet..

[44] Bernaciak J., Szczałuba K., Derwińska K., Wiśniowiecka-Kowalnik B., Bocian E., Sasiadek M.M., Makowska I., Stankiewicz P., Smigiel R. (2008). Clinical and molecular-cytogenetic evaluation of a family with partial Jacobsen syndrome without thrombocytopenia caused by an approximately 5 Mb deletion del(11)(q24.3). Am. J. Med. Genet. Part A.

[45] Favier R., Akshoomoff N., Mattson S., Grossfeld P. (2015). Jacobsen syndrome: Advances in our knowledge of phenotype and genotype. Am. J. Med. Genet. Part C Semin. Med. Genet..

[46] Walenkamp M.J., de Muinck Keizer-Schrama S.M., de Mos M., Kalf M.E., van Duyvenvoorde H.A., Boot A.M., Kant S.G., White S.J., Losekoot M., Den Dunnen J.T. (2008). Successful long-term growth hormone therapy in a girl with haploinsufficiency of the insulin-like growth factor-I receptor due to a terminal 15q26.2->qter deletion detected by multiplex ligation probe amplification. J. Clin. Endocrinol. Metab..

[47] Uddin M., Unda B.K., Kwan V., Holzapfel N.T., White S.H., Chalil L., Woodbury-Smith M., Ho K.S., Harward E., Murtaza N. (2018). OTUD7A Regulates Neurodevelopmental Phenotypes in the 15q13.3 Microdeletion Syndrome. Am. J. Hum. Genet..

[48] Moreno-De-Luca A., Helmers S.L., Mao H., Burns T.G., Melton A.M.A., Schmidt K.R., Fernhoff P.M., Ledbetter D.H., Martin C.L. (2011). Adaptor protein complex-4 (AP-4) deficiency causes a novel autosomal recessive cerebral palsy syndrome with microcephaly and intellectual disability. J. Med. Genet..

[49] Burgess T., Brown N.J., Stark Z., Bruno D.L., Oertel R., Chong B., Calabro V., Kornberg A., Sanderson C., Kelly J. (2014). Characterization of core clinical phenotypes associated with recurrent proximal 15q25.2 microdeletions. Am. J. Med. Genet. Part A.

[50] Shinawi M., Liu P., Kang S.H., Shen J., Belmont J.W., Scott D.A., Probst F.J., Craigen W.J., Graham B.H., Pursley A. (2010). Recurrent reciprocal 16p11.2 rearrangements associated with global developmental delay, behavioural problems, dysmorphism, epilepsy, and abnormal head size. J. Med. Genet..

[51] Goto T., Aramaki M., Yoshihashi H., Nishimura G., Hasegawa Y., Takahashi T., Ishii T., Fukushima Y., Kosaki K. (2004). Large fontanelles are a shared feature of haploinsufficiency of RUNX2 and its co-activator CBFB. Congenit. Anom..

[52] Khan A., Hyde R.K., Dutra A., Mohide P., Liu P. (2006). Core binding factor beta (CBFB) haploinsufficiency due to an interstitial deletion at 16q21q22 resulting in delayed cranial ossification, cleft palate, congenital heart anomalies, and feeding difficulties but favorable outcome. Am. J. Med. Genet. Part A.

[53] Meyer R.E., Liu G., Gilboa S.M., Ethen M.K., Aylsworth A.S., Powell C.M., Flood T.J., Mai C.T., Wang Y., Canfield M.A. (2016). Survival of children with trisomy 13 and trisomy 18: A multi-state population-based study. Am. J. Med. Genet. Part A.

[54] Wieczorek D., Newman W.G., Wieland T., Berulava T., Kaffe M., Falkenstein D., Beetz C., Graf E., Schwarzmayr T., Douzgou S. (2014). Compound heterozygosity of low-frequency promoter deletions and rare loss-of-function mutations in TXNL4A causes Burn-McKeown syndrome. Am. J. Hum. Genet..

[55] Jana B., Khanfar A., Ninan M. (2014). Durable hematological and major cytogenetic response in a patient with isolated 20q deletion myelodysplastic syndrome treated with lenalidomide. Case Rep. Oncol. Med..

[56] Shiels A., Bennett T.M., Knopf H.L., Yamada K., Yoshiura K., Niikawa N., Shim S., Hanson P.I. (2007). CHMP4B, a novel gene for autosomal dominant cataracts linked to chromosome 20q. Am. J. Hum. Genet..

[57] Schwarz K., Iolascon A., Verissimo F., Trede N.S., Horsley W., Chen W., Paw B.H., Hopfner K.P., Holzmann K., Russo R. (2009). Mutations affecting the secretory COPII coat component SEC23B cause congenital dyserythropoietic anemia type II. Nat. Genet..

[58] Yagi H., Furutani Y., Hamada H., Sasaki T., Asakawa S., Minoshima S., Ichida F., Joo K., Kimura M., Imamura S. (2003). Role of TBX1 in human del22q11.2 syndrome. Lancet (Lond. Engl.).

[59] Sullivan K.E. (2019). Chromosome 22q11.2 deletion syndrome and DiGeorge syndrome. Immunol. Rev..

[60] Rauch R., Hofbeck M., Zweier C., Koch A., Zink S., Trautmann U., Hoyer J., Kaulitz R., Singer H., Rauch A. (2010). Comprehensive genotype-phenotype analysis in 230 patients with tetralogy of Fallot. J. Med. Genet..

[61] Kharbanda M., Pilz D.T., Tomkins S., Chandler K., Saggar A., Fryer A., McKay V., Louro P., Smith J.C., Burn J. (2017). Clinical features associated with CTNNB1 de novo loss of function mutations in ten individuals. Eur. J. Med. Genet..

