# Supplementary material for: Identification of Extremely Rare Pathogenic CNVs by Array CGH in Saudi Children with Developmental Delay, Congenital Malformations, and Intellectual Disability
Source: Children (Basel). 2023 Mar 31;10(4):662. doi: 10.3390/children10040662 (PMC10136417; doi:10.3390/children10040662)
Supplement: Supplementary file 1 [file children-10-00662-s001.zip › children-2153985-supplementary.pdf]

Table S1. Clinical information of pediatric developmental delay, congenital malfunction, and intellectual disability patients included in this study.

S. No	Biobank Code	Gender	Age	Array Type	Symptoms/Phenotypes
1	BL-022-12	Female	5 years	CGH 1 × 244 A	Dysmorphic Feature
2	BL-025-14	Male	6.8 years	CGH2 × 400 k	Intellectual Disability, Congenital Heart Disease, Hypospadias, Dysmorphic Feature
3	BL-034-13	Female	3 years	CGH2 × 400 k	Central Hypotonia, Microcephaly
4	BL-047-13	Male	1.8 years	CGH2 × 400 k	Congenital heart disease, Dysmorphic Feature
5	BL-080-12	Male	4 years	CGH2 × 400 k	Delayed Speech, Dysmorphic Features
6	BL-099-12	Male	2 years	CGH2 × 400 k	Dysmorphic Feature, Intellectual Disability
7	BL-104-13	Male	1 years	CGH2 × 400 k	Hypotonia, Undescended Testis, Talipes, High Arched Palate
8	BL-144-12	Female	4 years	CGH2 × 400 k	Obesity, Congenital Heart Disease, Normal Milestones
9	BL-161-14	Female	1 years	CGH2 × 400 k	VSD, Failure to thrive
10	BL-168-13	Male	6 years	CGH 1 × 244 A	Dysmorphic Features
11	BL-181-13	Female	2.6 years	CGH2 × 400 k	Hypotonia, Cleft Lip, VSD, ASD, Pulmonary Stenosis, Dysmorphic, Delayed Speech, Not Walking
12	BL-186-12	Male	1.7 years	CGH2 × 400 k	Dysmorphic feature
13	BL-208-13	Female	2.6 years	CGH2 × 400 k	Spinal Muscular Atrophy.
14	BL-209-13	Male	7.9 years	CGH 1 × 244 A	Fragile X Syndrome, Autism
15	BL-210-12	Female	6 years	CGH 1 × 244 A	Delayed Speech
16	BL-211-14	Male	5 years	CGH2 × 400 k	Dysmorphic feature
17	BL-224-12	Male	2.3 years	CGH2 × 400 k	Microcephaly, Dysmorphic, Brain Atrophy
18	BL-346-12	Male	5 years	CGH2 × 400 k	Delayed Speech, Dysmorphic feature
19	BL-363-12	Male	2 years	CGH2 × 400 k	Delayed Speech, Dysmorphic feature
20	BL-376-13	Male	9 years	CGH 1 × 244 A	Hypogonadism, Microcephaly, Prader Willi Syndrome
21	BL-394-12	Male	4.7 years	CGH2 × 400 k	Dysmorphic feature
22	BL-401-13	Female	8 days	CGH 1 × 244 A	Congenital Heart Disease, Dysmorphic feature
23	BL-411-12	Female	5 years	CGH 1 × 244 A	Dysmorphic feature
24	BL-422-12	Female	8 years	CGH2 × 400 k	Intellectual Disability, Delayed Speech,
25	BL-437-11	Male	4 years	CGH2 × 400 k	Delayed Walking, Unstable, Muscle Wasting
26	BL-457-12	Male	2 years	CGH 1 × 244 A	DD, not walking, no speech, dysmorphic, hypotonia, epilepsy, microcephaly, preterm birth at 2 weeks, VSD at birth
27	BL-461-12	Male	4 months	CGH 1 × 244 A	Dysmorphic Feature, Small for date, DD, failure to thrive, small finger has two phalanges, simian crease in left hand
28	BL-464-12	Female	1.5 years	CGH2 × 400 k	Short Stature
29	BL-504-13	Male	1.6 years	CGH 1 × 244 A	Dysmorphic, Brain Atrophy, Muscle Dystrophy, Not Walking, No Speech
30	BL-518-13	Female	8 years	CGH2 × 400 k	Congenital Heart Disease
31	BL-525-13	Female	3.4 years	CGH2 × 400 k	Delayed Speech, Delayed Walking, Hypotonia,
32	BL-597-12	Female	1 month	CGH2 × 400 k	Dysmorphic feature, Low-set Ears, Closed VSD
33	BL-611-12	Male	6 months	CGH2 × 400 k	Hypotonia
34	BL-622-12	Male	7 years	CGH 1 × 244 A	Dysmorphic feature

35	BL-628-12	Female	29 days	CGH2 × 400 k	Ambiguous Genitalia, Dysmorphic
36	BL-642-12	Male	6 years	CGH2 × 400 k	Fragile X syndrome
37	BL-664-11	Female	10 months	CGH2 × 400 k	Congenital Heart Disease
38	BL-674-11	Female	2 years	CGH2 × 400 k	Developmental Delay
39	BL-681-12	Female	3.6 years	CGH2 × 400 k	Delayed Speech, Dysmorphic Feature, Mental Disability
40	BL-683-12	Male	4.7 years	CGH2 × 400 k	Dysmorphic feature
41	BL-723-13	Male	8 months	CGH2 × 400 k	GH Deficiency
42	BL-793-13	Male	3 months	CGH2 × 400 k	Congenital heart disease, DiGeorge Syndrome
43	BL-853-12	Female	1.7 years	CGH2 × 400 k	Congenital Heart Disease, ASD, PWS
44	BL-900-12	Male	8 months	CGH2 × 400 k	Hirschsprung Disease, Dilated Lat. Ventricle
45	BL-901-12	Male	9.5 years	CGH2 × 400 k	Hypospadias, Undescended Testis, VSD, Dysmorphic features
46	BL-902-10	Male	16 years	CGH2 × 400 k	Microcephaly, Intellectual Disability, Dysmorphic
47	BL-902-12	Male	4.5 years	CGH2 × 400 k	Dysmorphic features, Delayed Speech,
48	BL-954-12	Female	6 years	CGH2 × 400 k	Bilateral disease, Talipes, Microcephaly
49	BL-982-12	Male	8 years	CGH2 × 400 k	Intellectual Disability, Delayed Speech, Delayed Walking
50	BL-983-12	Female	2 years	CGH2 × 400 k	Not Walking, No speech
51	BL-1019-12	Male	3 years	CGH2 × 400 k	No Walking, Delayed Speech, Seizures
52	BL-1020-12	Male	3 years	CGH2 × 400 k	Floppy, Dysmorphic, Left Axial Polydactyly
53	BL-1042-12	Female	5.9 years	CGH2 × 400 k	Delayed Speech, Microcephaly
54	BL-1083-10	Female	7 months	CGH2 × 400 k	Hypotonia, Microcephaly
55	BL-1086-11	Male	6 years	CGH2 × 400 k	Intellectual Disability, ADHD, Dysmorphic, Seizures, Small ears, Triangular face, VSD at birth, No Speech, Delayed Walking, Epilepsy
56	BL-1087-11	Male	12 years	CGH2 × 400 k	Dysmorphic, Hyperactive, Intellectual Disability, ADHD, Small Ears, Seizures, No Speech, Delayed Walking
57	BL-1123-12	Male	2.5 years	CGH2 × 400 k	Delayed Speech, Floppy baby syndrome, Dysmorphic Features, Left Axial Polydactyly
58	BL-1138-12	Female	5.6 years	CGH2 × 400 k	Delayed Speech, Microcephaly, Brain Atrophy, Seizures
59	BL-1140-11	Female	5 years	CGH2 × 400 k	Dysmorphic features, VSD, Intellectual Disability
60	BL-1178-12	Female	5 years	CGH2 × 400 k	Delayed Speech
61	BL-1288-13	Male	6 years	CGH2 × 400 k	Intellectual Disability, IQ: 65, Microcephaly, Autism, Delayed speech, Hyperactive, Delayed Walking, slightly overweight, Abnormal Teeth, Ataxic Gait
62	BL-AM-14	Male	5 years	CGH2 × 400 k	Developmental Delay
63	BL-OY-14	Male	6 years	CGH2 × 400 k	Skeletal and cardiac abnormalities, Marfan Syndrome
